# Supplementary figures and images for: Delay-period activity in frontal, parietal, and occipital cortex tracks noise and biases in visual working memory
Source: PLoS Biol. 2020 Sep 8;18(9):e3000854. doi: 10.1371/journal.pbio.3000854 (PMC7500688; doi:10.1371/journal.pbio.3000854)

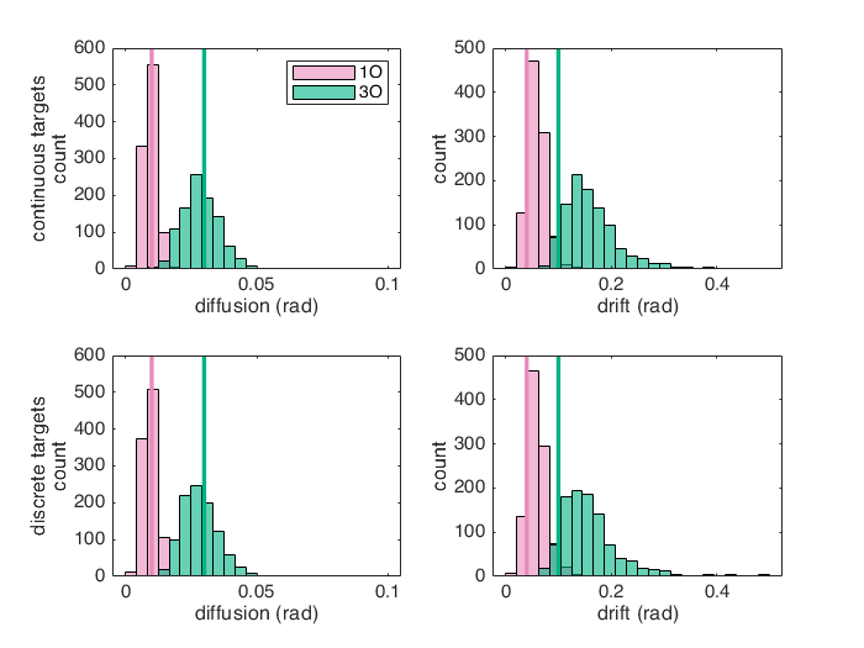

Supplement: S1 Fig — Pink: 1O condition. Green: 3O condition. Note that although we observe some bias in the estimate of drift when load = 3 due to the relatively modest number of trials per condition (50), the models fit to the empirical data nevertheless capture behavioral well (S3A Fig) and because this bias is a constant factor in our analyses across regions it cannot explain our neural results. Data are available at osf.io/ajq3z. 1O, 1 orientation; 3O, 3 different orientations. (TIF) [file pbio.3000854.s001.tif]

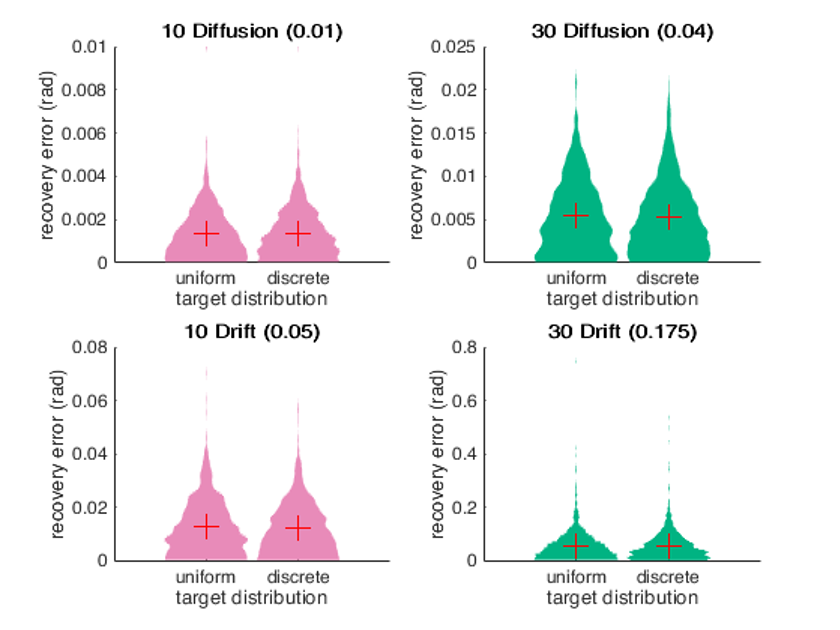

Supplement: S2 Fig — Violin plots show distribution over 1,000 simulated datasets. Red crosses indicate mean values. Pink: 1O condition. Green: 3O condition. Data are available at osf.io/ajq3z. 1O, 1 orientation; 3O, 3 different orientations; DDM, drift–diffusion model. (TIF) [file pbio.3000854.s002.tif]

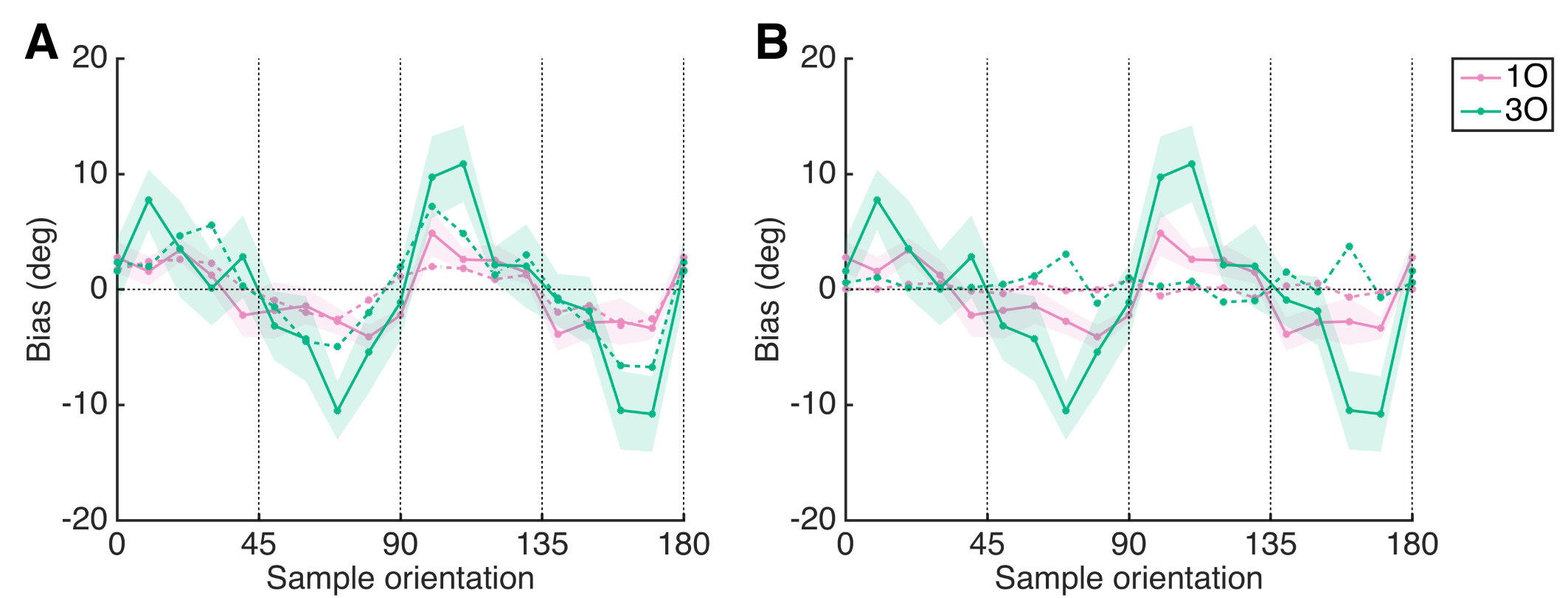

Supplement: S3 Fig — Sample orientations were categorized in 10° bins. Solid lines demonstrate the experimental data (shaded areas indicate ± 1 SEM), and dashed lines demonstrate model fits. A. behavioral data with DDM model fits. B. behavioral data with DOM model fits. Data are available at osf.io/ajq3z. 1O, 1 orientation; 3O, 3 different orientations; DDM, drift–diffusion model; DOM, diffusion-only model. (TIF) [file pbio.3000854.s003.tif]

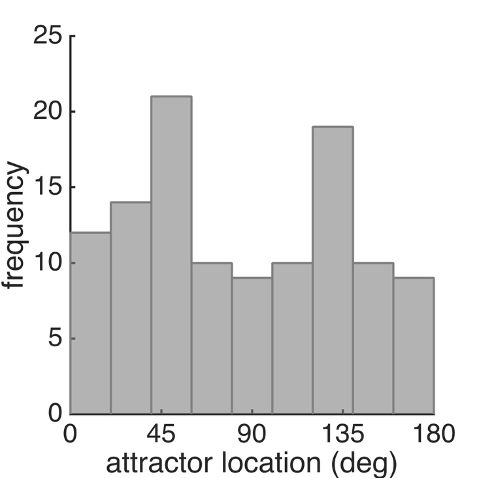

Supplement: S4 Fig — Data are available at osf.io/ajq3z. (TIF) [file pbio.3000854.s004.tif]

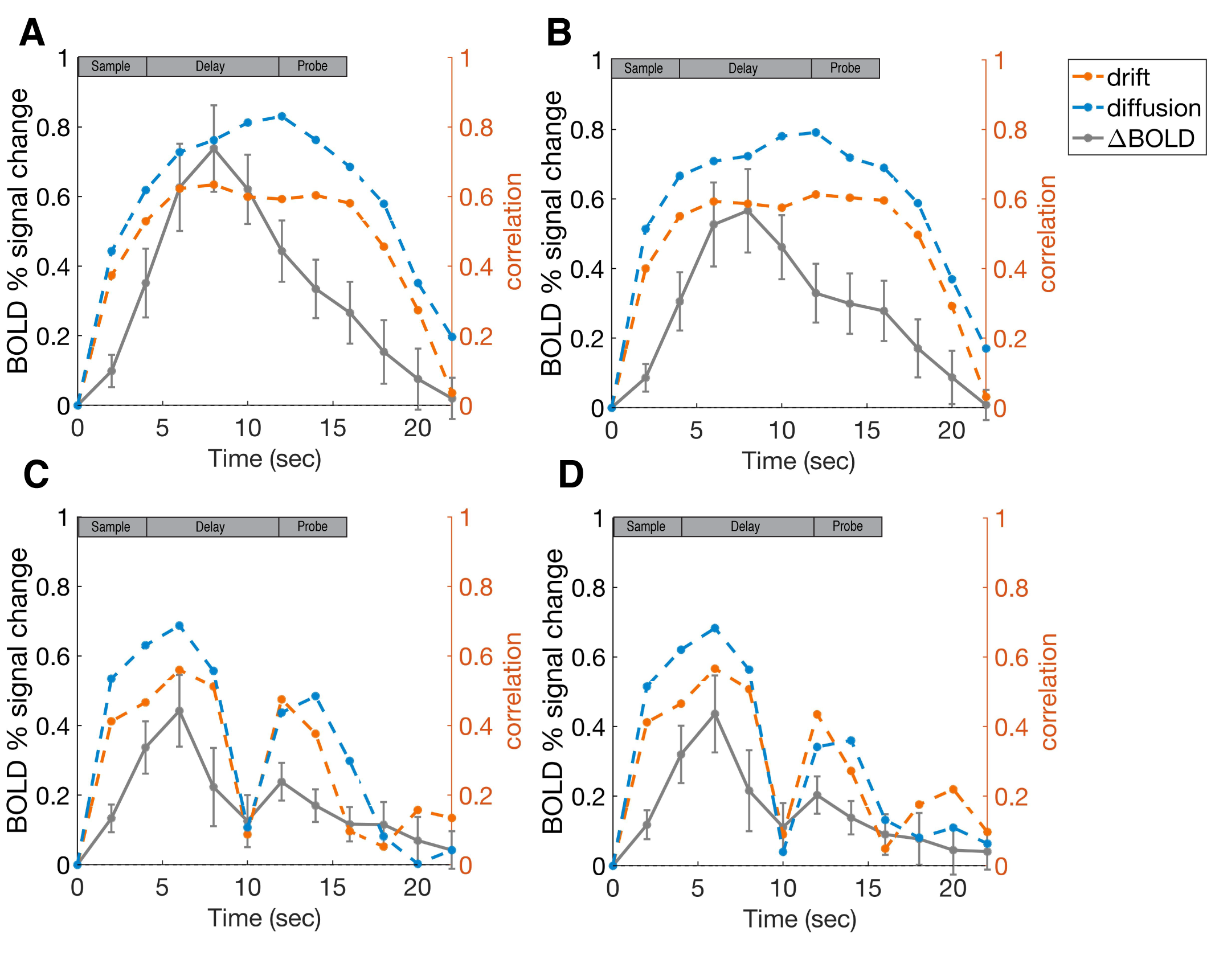

Supplement: S5 Fig — Error bars indicate ± 1 SEM. A. IPS. B. PFC. C. LO1. D. LO2. Data are available at osf.io/ajq3z. 1O, 1 orientation; 3O, 3 different orientations; IPS, intraparietal sulcus; LO, lateral occipital cortex; PFC, prefrontal cortex. (TIF) [file pbio.3000854.s005.tif]

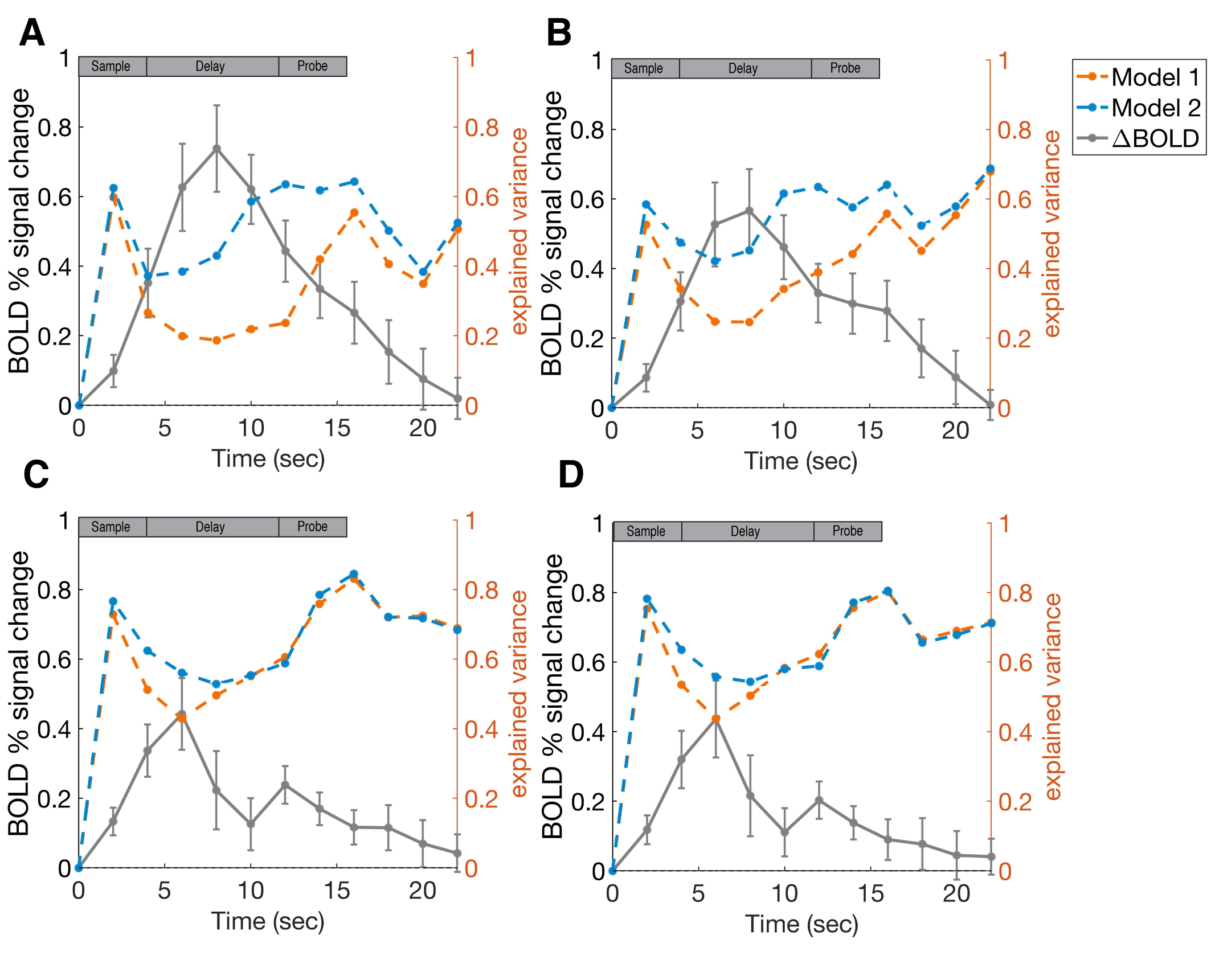

Supplement: S6 Fig — Positive difference in correlation indicates higher correlation for diffusion, and negative difference indicates higher correlation for drift. A. IPS. B. PFC. C. LO1. D. LO2. Data are available at osf.io/ajq3z. 1O, 1 orientation; 3O, 3 different orientations; IPS, intraparietal sulcus; LO, lateral occipital cortex; PFC, prefrontal cortex. (TIF) [file pbio.3000854.s006.tif]

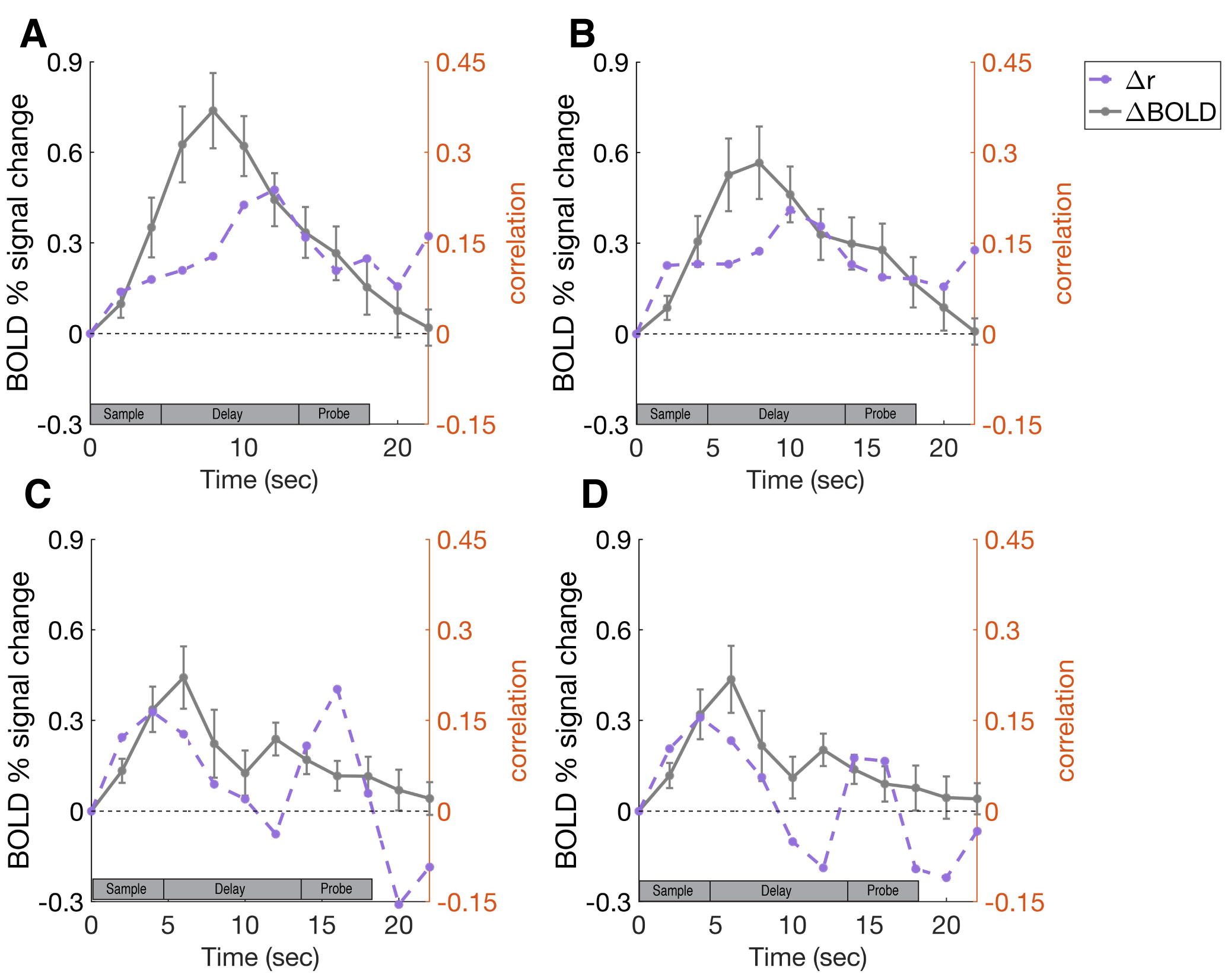

Supplement: S7 Fig — Error bars indicate ± 1 SEM. A. IPS. B. PFC. C. LO1. D. LO2. Data are available at osf.io/ajq3z. 1O, 1 orientation; 3O, 3 different orientations; IPS, intraparietal sulcus; LO, lateral occipital cortex; PFC, prefrontal cortex. (TIF) [file pbio.3000854.s007.tif]

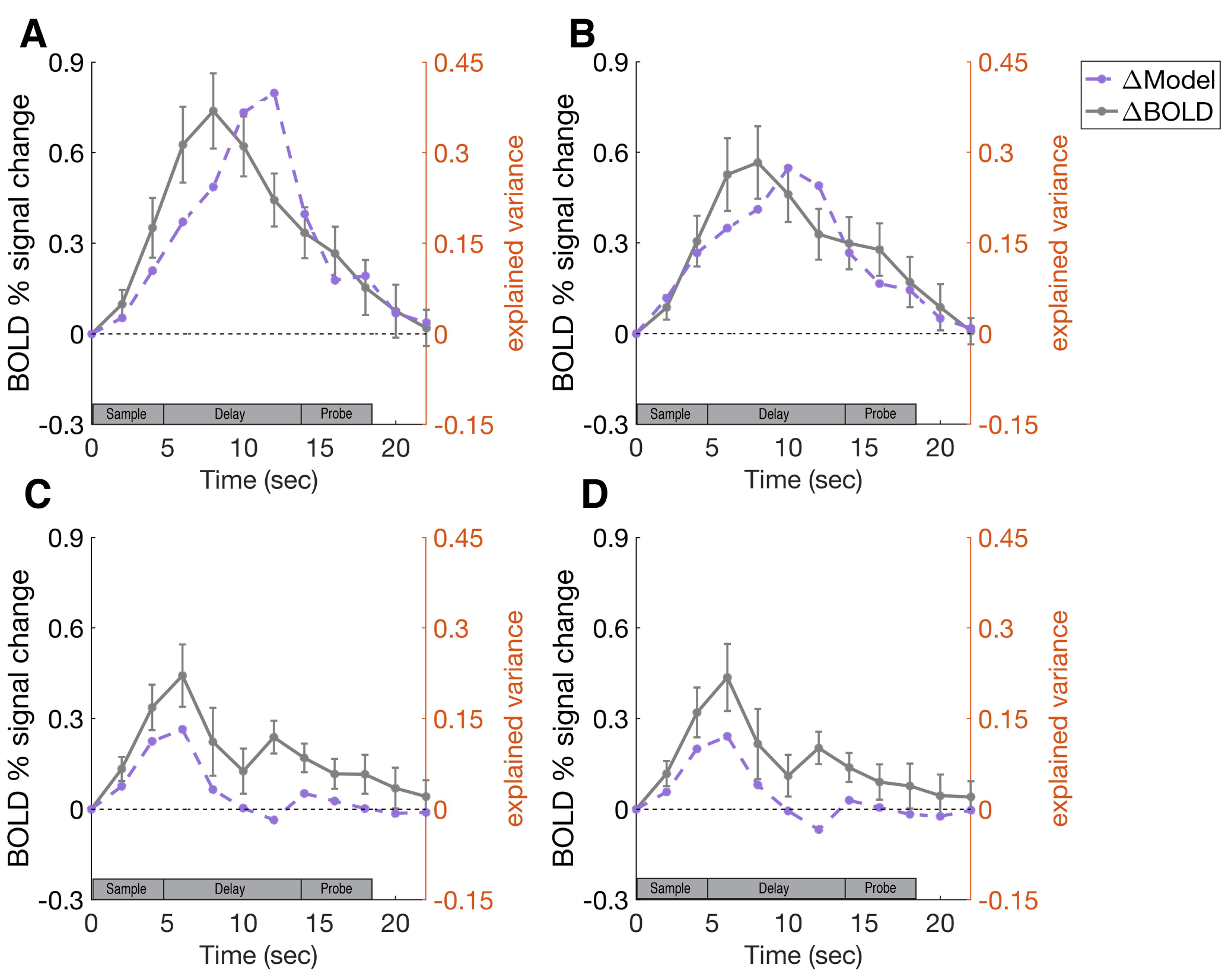

Supplement: S8 Fig — Positive difference in explained variance higher model fit for Model 2 (diffusion model), and negative difference indicates higher model fit for Model 1 (drift model). A. IPS. B. PFC. C. LO1. D. LO2. Data are available at osf.io/ajq3z. 1O, 1 orientation; 3O, 3 different orientations; IPS, intraparietal sulcus; LO, lateral occipital cortex; PFC, prefrontal cortex. (TIF) [file pbio.3000854.s008.tif]
