# Supplementary material for: Delay-period activity in frontal, parietal, and occipital cortex tracks noise and biases in visual working memory
Source: PLoS Biol. 2020 Sep 8;18(9):e3000854. doi: 10.1371/journal.pbio.3000854 (PMC7500688; doi:10.1371/journal.pbio.3000854)
Supplement: Attachment — Submitted filename: DAMCorr_ResponseLetter_Final.docx [file pbio.3000854.s009.docx]

**Point-by-point response to PBIOLOGY-D-20-00344R1**

We would like to thank the reviewers for their insightful and constructive comments. To address the reviewers’ concerns, we have conducted additional analyses, and included relevant information in the main text and/or supporting information of the revised manuscript. We have also modified some of our claims in Discussion, following the reviewers’ suggestions. Below we have reprinted the reviewers’ comments *in italicized Times New Roman*, and inserted our point-by-point responses in sans-serif Arial.

*Reviewer #1: Synopsis: Yu et al. use a novel modeling approach to estimate parameters for bias and variance in an orientation recall task, then examine how these parameters co-vary with memory-load-dependent changes in frontoparietal BOLD activity during the delay period of the task. They find that load-dependent changes in frontoparietal activity are correlated with both variance and bias, with the former explaining a larger proportion of variance than the latter. Moreover, they report that overall activation in lateral occipital cortex is best predicted by bias.

Evaluation: This paper makes novel use of a new model that provides a good description of working memory performance in humans and monkeys. Determining the why and how of load-dependent changes in delay period activity is an important goal - dozens if not hundreds of studies have documented load-dependent changes in activation during WM task, yet a detailed explanation for these changes is lacking. I have two major concerns about this paper. The first is technical and focuses on whether it's fair for the authors to apply their DDM model to the current data set. The second is conceptual and focuses on what these data tell us about the structure of working memory or models thereof. I'm happy to clarify my comments and/or have a dialogue with the authors if it'd be helpful.

Best wishes,

Edward Ester, Florida Atlantic University

Majors:

1.      Is it fair to apply the DDM to these data? As I understand it, the DDM used by the authors is constructed to explain bias and variance in subjects' responses under the assumption that to-be-reported items are uniformly sampled from a fixed parameter space - in this case, orientations on the interval [0,pi). Yet the orientations sampled here were preferentially drawn from subdistributions centered on 9 orientations (aside: was the jitter added to each orientation on each trial uniformly or normally distributed? I couldn't find this information in the manuscript). This creates two potential problems: the first and largest concern is that nonuniform sampling it could encourage bias in participants' orientation reports towards the nine "pedestal" orientations - e.g., participants are more likely to report a 23° stimulus as 20° because they've repeatedly seen exemplars drawn from a distribution centered on 20° - and this would result in biased parameter estimates. The second concern is that even if one assumes that participants' reports track the actual target values on each trial, it's not clear whether or how well the Panichello et al. model works in a nonuniform stimulus space. For example, does the model require uniform sampling? Does nonuniform sampling introduce additional bias or variance into parameter estimates? This absolutely needs to be worked out (and could presumably be done via simulation); otherwise it's very difficult to accurately interpret correlations between model parameters and BOLD data.*

**Response**:

We thank the reviewer for raising this important point. To quickly address the aside and to clarify the stimuli used in the experiment: the random jitter added to each stimulus was uniformly distributed between ±0-5°. Besides this random jitter on each trial, we also added a jitter of 1-10° to each subject’s nine target orientations (from 0° to 160° in 20° increments), so that each subject viewed slightly different target orientations (e.g., from 1° to 161° in 20° increments, or from 5° to 165° in 20° increments, etc). We have clarified this information on lines 304-306 in Methods.

We agree with the reviewer that there are two important questions here that we need to address: nonuniform sampling and discrete target values.

Regarding the first question, i.e., “whether non-uniform sampling would result in participants’ biased reports towards the nine target orientations”, we believe this is less of a concern for the current study, because the purpose of this study is to compare the amount of drift and diffusion between two memory load conditions. Therefore, even if, for example, participants’ reports were biased towards 20° because 20° was one of the nine target orientations, the same bias should have influenced both load conditions, and therefore any difference between 1O and 3O should have been caused by the difference in memory load, rather than the value of 20° per se. In other words, the purpose of this study was not to characterize the absolute locations of the attractors, which are likely to change when environmental statistics change (see Panichello, DePasquale, Pillow, & Buschman, 2019 for colors; and Taylor & Bays, 2018 for a similar idea in orientations), but to characterize changes in the amount of attractor biases across different experimental conditions. Therefore, non-uniform sampling shouldn’t affect the comparison of parameters between 1O and 3O. Furthermore, to fully address the reviewer’s concern, we plotted subjects’ response bias as a function of distance towards the bin centers (i.e., trialwise jitter, from -5° to +5° in steps of 1°), averaged across bin centers and subjects. To elaborate, for each trial, the sample orientation was (one of the) bin center ± 0-5°. If there were an attractor bias towards the bin center, one should expect a negative slope passing through 0 (i.e., bin center), meaning that subjects’ responses were biased towards the bin center, when they remembered orientations that were ± 1-5° away from the bin center. However, we did not observe such a pattern in Response Figure 1, suggesting influences from the target orientations were minimal, if not non-existent.

**Response Figure 1**. Response bias as a function of sample jitter for 1O and 3O conditions (n = 30). 0° represents bin centers (collapsed across nine orientations). shaded areas indicate ± 1 SEM.

Second, to illustrate whether the DDM could be successfully fit to datasets with discrete target values, we generated simulated data from the DDM using known parameters and tested the ability of our fitting procedure to recover these parameters when targets in these simulated datasets were discrete and uniform. Simulated datasets were matched to the experimental behavioral datasets: each consisted of 50 1O and 50 3O trials with an 8-second delay. The orientation of targets and non-targets were either: (1) drawn from a pool of 9 orientations ranging from 0° to 160° in 20° increments with a random jitter of 1-10° added to each subject’s targets and ± 0-5° added to stimulus on each trial (discrete condition); or (2) drawn uniformly from values ranging from 0 to 180° (continuous condition). The parameters of the model were set to be similar to those observed empirically: 1O drift, 1O diffusion, 3O drift, and 3O diffusion were set to 0.04, 0.01, 0.10, and 0.03, respectively. The drift function was parameterized such that values were biased towards approximately 45° and 135°, consistent with our observations here and with previous reports in the literature. For each trial, a simulated report was drawn from the probability distribution over reports generated by the model (given the chosen parameters, target value, non-target values, delay, and set size).

1,000 discrete and 1,000 continuous datasets with randomly generated target and non-target values were created. For each dataset, we fit the DDM model and recovered the maximum likelihood parameters, exactly as for the empirical data, and compared the recovered parameters to the known, generative parameters (Response Figures 2 and 3).

The distribution of target parameters did not significantly impact the ability to recover these known parameters. The bias and variance of the estimates were similar across the uniform and discrete condition (Response Figure 2). Note we observed a small bias in estimates for larger drift values, driven by the modest number of trials per condition (50). However, this bias is constant for all of our comparisons across ROIs, and so cannot explain the differences across regions reported in the main text. Critically, mean recovery error did not significantly differ between the uniform and discrete condition for any of the parameters (all *p*s > 0.4, bootstrap). Therefore, discrete target selection does not influence the model fits of DDM. We have included these results on lines 381-400 in the main text and in new figures (S1 Fig and S2 Fig) in the supporting information.

**Response Figure 2**. Distribution of recovered parameters across 1,000 simulated datasets (histogram) and generative values (lines). Pink: 1O condition. Green: 3O condition.

**Response Figure 3**. Mean recovery error for known generative parameters of the DDM (values in parentheses). Violin plots show distribution over 1,000 simulated datasets. Red crosses indicate mean values. Pink: 1O condition. Green: 3O condition.

*2.      Setting aside #1, I'm having a hard time understanding the implications of the study. The key points are that model estimates of variance correlate with load-dependent changes in the BOLD signal in frontoparietal cortex, and that model estimates of bias correlate with load-dependent changes in lateral occipital cortex. What are the implications of these findings? For example, some of the authors on this paper have previously advocated for a model where frontoparietal cortical areas coordinate memory storage in posterior sensory areas. On that account, I would've predicted frontoparietal areas to correlate more strongly with bias and visual areas to correlate with variance (e.g., frontoparietal areas implement bias to account for more variability in sensory areas during higher memory loads). As-is, I'm not sure what the current findings tell us about the architecture of working memory, other than that different brain areas correlate more strongly with different latent parameters. Some additional context and explanation is necessary.*

**Response**:

We agree that more discussion on the implications of this work is needed. In our revision we now emphasize that the important implications of this work are: (1) to add some specificity to this “frontoparietal = coordination/occipital=storage” perspective; and (2) to address the fact (long known to cognitive psychology) that storage entails more than just the retention of a high-fidelity sensory representation. Relevant information has been incorporated into Discussion on lines 224-270 of the revised manuscript.

With regard to (1), we have previously reported that delay-period activity in frontoparietal cortex is more sensitive to manipulation of a putatively control-related factor (context binding) (Gosseries et al., 2018), but this interpretation relied on the validity of our assumptions about the intuited cognitive processes indexed by these manipulations. In the current study, in contrast, we use a quantitative method grounded in dynamical systems theory to decompose memory errors into two sources that have principled definitions -- drift and diffusion -- and then show (for the first time) that frontoparietal load-sensitive activity relates more strongly to diffusion than to drift. Therefore, delay-period activity in frontoparietal cortex likely reflects the engagement of control processes in response to load-related increases in diffusive noise.

With regard to (2), our results in occipital cortex suggest important commonality with other demonstrations of bias in visual cognition, such as the influence of prior expectation on the representation of motion (Kok, Brouwer, van Gerven, & de Lange, 2013), and the influence of learning on population-level representations of category boundaries in visual cortex (Ester, Sprague, & Serences, 2020). Although we initially shared the reviewer’s intuition, we would suggest that the fact that these results are at odds with this prediction demonstrates their novelty and potential impact. Furthermore, because our current study did not involve a learning intervention (unlike the Kok and Ester studies), an additional novel implication is its suggestion that what we already know about the neural bases of the biasing effects of recent experience on visual cognition may extend to more trait-like “pre-existing” attractor landscapes that have been sculpted through a lifetime of experience with the visual world.

It is important to note that the patterns in our results were not absolute; for example, we also found a significant correlation between load-sensitive drift and BOLD activity in frontoparietal cortex. The critical comparison was which parameter explained *more* variance in BOLD data. Therefore, it is likely that occipital and frontoparietal regions are both implicated in the generation and processing of drift and diffusion in human working memory performance, but that the functioning of frontoparietal cortex is less influential on load-related increases in drift (i.e., bias) than it is on load-related increases in diffusion. Moreover, bias-related processes in frontoparietal and occipital cortex may play different roles in behavior, with the former reflecting control processes related to implementation of biases, and the latter reflecting stimulus-specific representations that are biased towards the attractors.

Lastly, following Reviewer 2’s suggestion, we have toned down our interpretations of results from LO, because some of these effects were only trending. Nonetheless, we believe that a strong effect for diffusion in frontoparietal cortex, and a lack (or even reversal) of such an effect in LO, provide a richer understanding of the dissociation of functions of these brain regions in working memory maintenance.

*Minors:

1.      If space allows, it'd be helpful to include a brief introduction to the drift-diffusion model (DDM) in the main text and a mathematical derivation in the methods. I hadn't read the Panichello et al. paper prior to reviewing this study (though it's been on my "reading list" for a while), and the term "drift-diffusion model" has a storied history in the perceptual decision making literature (e.g., Ratcliff and others). Initially I thought the authors were going to be modeling forced-choice reaction time data and mapping those parameters on to BOLD activation, which led to some considerable head-scratching during my first read of the introduction and results.*

**Response**:

We thank the reviewer for the suggestion, and we have added a brief introduction of the drift-diffusion model to the beginning of the Result section on lines 100-104 to avoid confusion:

“We fit data from the behavioral session using a discrete attractor model (Panichello et al., 2019). This circular drift-diffusion model (DDM) fits the dynamic evolution of memories with two distinct processes: random noise (*diffusion*); and systematic *drift* towards one of several stable attractors. Notably, when the drift parameter is removed, the remaining diffusion-only model (DOM) is equivalent to a classic mixture model.”

We have also updated corresponding text on lines 356-379 in Methods with the following:

“We fit the data from the behavioral session using a discrete attractor model (Panichello, et al. 2019). This model assumed that memories evolve over time according to two distinct processes: random noise (*diffusion*), and systematic *drift* towards attractor states in the stimulus space. Specifically, the temporal evolution of a remembered stimulus orientation θ is modeled using the partial differential equation:

$$d\theta=\beta_{L}G(\theta)dt + \sigma_{L}dW$$

where G is a function describing the direction and magnitude of drift across stimulus space, $\beta_{L}$ defines the gain of the drift for a given load *L*, and *W* is an additive white noise process with variance $\sigma_{L}$. Thus, $\beta_{L}G(\theta)dt$ describes the influence of drift and $\sigma_{L}dW$ the influence of random noise on memory. By analogy with the decision-making literature, in which drift-diffusion models are often used to model latent evidence for a behavioral choice in a linear 1D space, this drift-diffusion model (DDM) describes the latent value of the subject’s memory in a circular 1D space. Both parameters are rates, with a unit of rad/s indicating the rate of diffusion and the maximum instantaneous drift rate. Unlike the Panichello et al. (2019) study, here we fitted behavioral data without separating out encoding and delay processes, because the length of memory delays was not manipulated in this experiment.

The model also captures variance due to random responses and reports of non-target items (following Zhang and Luck, 2008, Bays et al., 2009, see Panichello et al., 2019 for details). We identified the maximum likelihood estimates of $\beta_{L}$ (drift) and $\sigma_{L}$ (diffusion) for each subject and analyzed these fit parameters as described in the main text. Notably, when the drift parameter is removed, the remaining diffusion-only model (DOM) is equivalent to a classic mixture model. The comparison between performance of the DDM and DOM models was evaluated by combining data from all subjects, and computing the average difference in cross-validated log-likelihood value across 10 folds.”

*2.      As Panichello et al. show, the DDM approach does a nice job of capturing biases in color report data. I'm less certain it generalizes to orientation, especially given that orientations weren't uniformly sampled in this experiment. For example, can the DDM capture well-known cardinal biases in delayed orientation reports (e.g., reported in multiple studies by Rademaker, Pratte, Landy, et al?). This strikes at the question of whether it's fair to model the current data with the DDM.*

**Response**:

The distribution of participants’ raw responses (as shown in Fig 1B) suggested that participants’ responses were indeed biased away from the cardinal orientations (0° and 90°; i.e., repulsive bias), and biased towards the oblique orientations in between the two cardinal orientations (attractive bias). To confirm that participants showed cardinal biases in the current study, we plotted participants’ recall error (the difference between response and sample) as a function of sample orientation (in 10° bins) as in previous studies. And consistent with previous reports, our participants’ orientation reports did show a clear bias away from the cardinal orientations that is dependent on the number of items held in working memory (Response Figures 4A and 4B; solid lines).

More importantly, when we plotted model predictions from the DDM and DOM as a function of sample orientation (model prediction was simulated by 1000 trials per subject to increase the stability of effects), we found that the DDM did a better job at capturing cardinal bias (Response Figure 4A, dashed lines) compared with the DOM (Response Figure 4B, dashed lines). Pearson correlation between actual and predicted data was positive and significant for both 1O (*r* = 0.840, *p* = 0.00007) and 3O (*r* = 0.876, *p* = 0.000001) with the DDM, but not with the DOM (1O: *r* = -0.080, *p* = 0.745; 3O: *r* = -0.315, *p* = 0.190).

**Response Figure 4**. Response bias as a function of sample orientation for 1O and 3O conditions (n = 30). Sample orientations were categorized in 10° bins. Solid lines demonstrate the experimental data (shaded areas indicate ± 1 SEM), and dashed lines demonstrate model fits. **A**. behavioral data with DDM model fits. **B**. behavioral data with DOM model fits.

Furthermore, the DDM returns an estimate of each participant’s fitted attractor locations. We plotted the distribution of fitted attractor locations across all participants (Response Figure 5). The histogram (from 0° to 180° in 20° bins) showed two clear peaks at oblique orientations near [40° 60°] as well as near [120° 140°]. In other words, the estimated attractor locations matched the center of biases, which is consistent with the observed response bias in Fig 1B and Response Figure 4. These results together suggest the DDM can indeed capture cardinal bias in orientations. We have included these results on lines 117-122 in the main text and figures (S3 Fig and S4 Fig) in supporting information.

**Response Figure 5**. Distribution of fitted attractor locations (in 20° bins; n = 30).

Lastly, we repeated the analyses on model-free analogues of drift and diffusion (bias and precision), to see if our main results could still hold without any model fitting of the data. Specifically, we categorized all samples into four bins, two cardinal bins in [67.5° 112.5°] and [157.5° 22.5°], and two oblique bins in [22.5° 67.5°] and [112.5° 157.5°]. Within each bin, we calculated the amount of repulsive/attractive bias from the bin center (i.e., 0° and 90° for the cardinal bins, 45° and 135° for the oblique bins), as well as the standard deviation of the recall error (i.e., error variability). We then averaged the bias (over absolute values) and variability across the four bins, and obtained model-free estimates of bias and variability. The two model-free estimates both increased with increasing memory load, similar to the model-based parameters (Response Figure 6A). Furthermore, within-subject correlation analysis yielded qualitatively similar results to those using model-based parameters from the DDM, and load-dependent changes in both model-free bias and model-free variability correlated with load-dependent changes in BOLD activity in both IPS (Response Figure 6B) and PFC (Response Figure 6C).

Together, these analyses confirmed that cardinal bias and load-dependent changes in bias and precision are inherent properties of the data, and that the DDM is good at capturing these characteristics of the orientation data.

**Response Figure 6**. Model-free measures. **A**. Model-free measure of bias and variability for 1O and 3O. **B**. Within-subject correlation between IPS BOLD and bias/variability. **C**. Within-subject correlation between PFC BOLD and bias/variability.

*Reviewer #2: Signed review by Ed Vogel

This article examines how a drift and diffusion model of within subject variation in working memory precision for orientation corresponds to delay period BOLD increases across cortex driven by item load (1 vs 3). The key finding is that while evidence for drift and diffusion were observed in frontal and parietal areas, diffusion explained most of the variance. By contrast, drift explained most of the variance in LO.

Overall, I think this is a very interesting and important study because it connects variation in WM behavioral precision to neural activity in a rigorous and model driven approach. The application of the DDM'd error performance to BOLD load activations is clever and novel.  I found that the study narrative is clear and easy to follow and that the frontal and parietal results are very compelling.

My chief concern is the interpretation of the LO results, most of which are "trending" in significance. I guess I'm not all that comfortable embracing the .08 as trending for drift while at the same time rejecting the .18 for diffusion. Of course, I think it is still important to report these results. However, I think some softening of the general conclusion that the results support a dichotomy is warranted.*

**Response**:

Although we demonstrated a reliable effect in LO using whole-brain analysis at the voxel level without predefining a specific LO ROI, and that drift did survive stepwise regression in LO, we agree with the reviewer that the results in LO were weaker than those in IPS and PFC as reflected in the marginally significant p-values in the within-subject correlation analysis. Furthermore, as indicated by our time course analysis (details to be found in our response to the next question), the LO results were also less robust in terms of temporal profile. Therefore, we have revised the manuscript, following the reviewer’s suggestion, to tone down our conclusion for LO, throughout the Discussion section. For example:

on lines 235-236: “…In comparison to IPS and PFC, although the effects were generally weaker, delay-period activity in LO showed an opposite trend…”

on lines 258-260: “…drift-dominant effects in LO were comparably weaker, and were only present during part of the trial (the delay and part of the probe period), compared to the larger-in-magnitude, trial-spanning diffusion-dominant effects in frontoparietal cortex...”

on lines 275-277: “…we see that stimulus-nonspecific factors, as reflected in the relationship between load-dependent changes in behavior (drift and diffusion) and delay-period activity, are also *suggestive* of such a distinction…”

*Perhaps related to the above point, I have some uncertainty about whether the smaller and shorted lived BOLD delay period signal in LO is impacting the fits to the behavioral models as compared to the larger longer lasting activation in PFC/PPC. More broadly, do increases in SNR (e.g., BOLD increase with load) equivalently impact the how the two models fare in predicting variance?*

**Response**:

We thank the reviewer for pointing this out. We agree that SNR is an important factor in determining the significance of the regression analysis, because we used the regression models to characterize load-dependent changes within subjects. In the extreme case where there is no difference in BOLD between loads, and/or no difference in behavioral performance between loads, there cannot be robust within-subject correlations.

However, we think SNR alone cannot explain all the effects observed in the current set of results. To demonstrate how increases in BOLD difference between 1O and 3O impacted the fits of the two models (Model 1: BOLD ~ drift (DDM) + subject; and Model 2: BOLD ~ diffusion (DDM) + subject), we performed the same regression analysis on all time points during the trial. We then plotted the within-subject correlations for drift and diffusion, against BOLD difference between the two memory loads, for every time point, in every ROI (Response Figure 7; and also see Response Figure 8 for a plot of difference in correlations). We also plotted the explained variance of Model 1 and Model 2, against BOLD difference between the two memory loads, for every time point, in every ROI (Response Figure 9; and also see Response Figure 10 for a plot of difference in explained variance). We found that although there was indeed a trend of larger difference in correlation and in model fits with higher SNR, the relationship between the two was not simply linear. For example, in IPS, maximum BOLD difference was at 8 s after trial onset, but maximum difference between the two correlations and between the model performances was at 12 s after trial onset (i.e., late-delay period, the time point of interest in the manuscript). Furthermore, 4 s and 12 s had comparable SNR in BOLD difference, but the difference in correlation and model performance at 12 s was significantly higher.

When comparing between regions, diffusion fits IPS BOLD better than drift throughout the entire trial, regardless of SNR; in LO2, however, drift fits better than diffusion only during the delay and part of the probe period. These differences in temporal profile again cannot be simply explained by differences in SNR.

Therefore, although we agree that SNR does have an important influence on model performance, we think that our observed difference between frontoparietal and occipital cortex cannot be simply explained by “higher SNR in frontoparietal cortex and lower SNR in occipital cortex”. We have included relevant results on lines 186-196 in the revised manuscript and figures (S5-8 Fig) in supporting information. Specifically, we have acknowledged this point on lines 260-264 in Discussion of the revised manuscript:

“Although this difference cannot by simply explained by a difference in BOLD activity between loads, as seen in the comparison of time courses of correlations, it remains to be examined whether this pattern would change, if, for example, a higher memory load was employed to increase load-dependent effect sizes in BOLD and behavior.”

**Response Figure 7**. Time course of difference in BOLD activity between 1O and 3O (difference is shown in absolute values regardless of the direction of difference; gray) and of within-subject correlation for drift (orange) and diffusion (blue; correlations are shown in absolute values for comparisons). Error bars indicate ± 1 SEM. **A**. IPS. **B**. PFC. **C**. LO1. **D**. LO2.

**Response Figure 8**. Same as Response Figure 7, except that difference in correlation (diffusion – drift, purple) was shown. Positive difference in correlation indicates higher correlation for diffusion, and negative difference indicates higher correlation for drift. **A**. IPS. **B**. PFC. **C**. LO1. **D**. LO2.

**Response Figure 9**. Time course of difference in BOLD activity between 1O and 3O (difference is shown in absolute values regardless of the direction of difference; gray) and of explained variance for Model 1 (orange) and Model 2 (blue). Error bars indicate ± 1 SEM. **A**. IPS. **B**. PFC. **C**. LO1. **D**. LO2.

**Response Figure 10**. Same as Response Figure 9, except that difference in explained variance (Model 2 – Model 1, purple) was shown. Positive difference in explained variance higher model fit for Model 2 (diffusion model), and negative difference indicates higher model fit for Model 1 (drift model). **A**. IPS. **B**. PFC. **C**. LO1. **D**. LO2.

*Reviewer #3: This study examines behavioral and fMRI data from an experiment testing working memory for orientation. The behavioral responses are fit with a recently-proposed dynamical model of working memory based on drift-diffusion to stable attractors (Panichello et al., 2019) and correlations are identified between these parameters and BOLD actiivity in different brain regions.

This is a novel and (in principle) promising approach, but unfortunately the study has a major flaw: although the model is dynamical the data is not, i.e. experimental delay time is fixed and the BOLD correlation analysis is based on averaging over a fixed time window. Because the drift-diffusion model is fit to a single timepoint, the diffusion component presumably is simply measuring mean variability of recall errors and the drift component measuring the strength of stimulus-specific biases. Although only target-averaged response distributions are plotted (in Fig 1B) the pattern is consistent with a repulsive bias away from the cardinal orientations. This is a well-documented phenomenon for orientation judgments, not just in short-term memory (e.g. Taylor & Bays, J Neurosci, 2018) but also in immediate perceptual judgments (e.g. Tommasini et al, Vis Res, 2010) with no memory component and no delay interval - implying that temporal drift is unlikely to be responsible. It is also questionable whether difusion makes much of a contribution to error variability in this data, with some studies claiming the effects of delay are weak to non-existent (e.g. Shin, Zou & Ma, J Vis, 2017). Perhaps the authors could consider a complete reframing of the paper in terms of brain regions that correlate with (direct measures of) error variability vs biases.*

**Response**:

We thank the reviewer for making this point. We agree that, because memory delay was not manipulated in the current study, our behavioral modeling results reflect influences from both encoding and maintenance of stimuli in working memory. We have included one paragraph on lines 264-270 in Discussion to acknowledge the limitation of our current design:

“It is also the case that, because our study did not manipulate the length of the delay period, influences from encoding- and delay-related processes on our estimates of drift and diffusion parameters thus cannot be dissociated. Therefore, our results should not be interpreted as being specific to the delay period, but rather as illustrating relations between delay-period neural activity and trialwise measures of drift and diffusion. Future studies using variable memory delays will be able to specifically relate drift and diffusion during encoding and memory to neural activity.”

Nevertheless, to ensure that our general approach is appropriate and to determine if parsing out drift and diffusion during encoding and memory will prove fruitful in future work, we analyzed data available on OSF from a published study (Rademaker, Park, Sack, & Tong, 2018), which involved a working memory task on orientations (n = 12, load = 1, delay varied in 1, 3, 6, 12 s). We modeled trials with a delay of 1 s and 6 s to separate out the encoding and maintenance processes, and to better match the time course of the current study. Note that this is a very conservative dataset to use for testing, because memory drift and diffusion are generally much weaker when load is 1, as shown for color (Panichello, DePasquale, Pillow, & Buschman, 2019). We compared this full model (including encoding drift, encoding diffusion, memory drift and memory diffusion), with two other models, one included only encoding drift and encoding diffusion (i.e., no memory components), and another included only encoding drift, encoding diffusion, and memory diffusion (i.e., no memory drift). Model comparison was performed using the cross-validated log likelihood method, as reported in the main text. We found that the full model outperformed both models (difference in cross-validated log likelihood: 85.6 between full model and no memory components model, and 3.9 between full model and no memory drift model). These results suggest that there’s indeed an effect of drift and diffusion during delay, albeit to a lesser degree.

In addition, as noted by the reviewer, although our behavioral data were not dynamic, our neural data were indeed dynamic because we measured BOLD activity for all time points during the trial. To examine whether the brain-behavior correlation we observed in the original manuscript was dynamic, we performed the same regression analysis on all time points during the trial. We then plotted the within-subject correlation for drift and diffusion, against BOLD difference between the two memory loads, for every time point, in every ROI (Response Figure 7; and also see Response Figure 8 for a plot of difference in correlations; please see figures in our response to Reviewer 2). We also plotted the explained variance of Model 1 and Model 2, against BOLD difference between the two memory loads, for every time point, in every ROI (Response Figure 9; and also see Response Figure 10 for a plot of difference in explained variance).

In both IPS and PFC where we observed an effect of ‘diffusion > drift’, we found that the effect (i.e., Model 2 > Model 1, or diffusion > drift) started to emerge at ~4 s after trial onset, and sustained until the end of the trial. Critically, the largest effect was in the late delay period (12 s), beyond what would be expected for an effect limited to encoding.

In both LO1 and LO2, however, the effect of ‘drift > diffusion’ started to emerge during the delay period (i.e., ~10-12 s), and also during part of the probe period (i.e., ~18-20 s). During the sample period, however, a ‘diffusion > drift’ effect was present (~4-8 s). In other words, the brain-behavior correlation was dynamic across the trial. Again, this is not consistent with only an effect during encoding (as that should not change over time).

In the revised manuscript, we continue to use the late-delay period to measure correlation between behavior and BOLD activity. This time period showed the most prominent effect for diffusion in frontoparietal cortex and for drift in LO, was closest to subjects’ behavioral response in time (considering a 4-6 s of hemodynamic delay in BOLD signals), and was less contaminated by sample- or response-related signals. In addition, in the revised manuscript, we have included the new analysis of the time course of correlation (on lines 186-196 in the main text with new figures in supporting information). Specifically, and following the suggestion of Reviewer 2 (on softening our conclusion for LO), we now acknowledge that diffusion played a dominant role in explaining BOLD activity in both IPS and PFC, and this effect was robust and sustained across the trial. In comparison, although a similar effect was observed for drift in LO, this effect was weaker, and was only present during the delay and probe periods.

**References**

Ester, E. F., Sprague, T. C., & Serences, J. T. (2020). Categorical Biases in Human Occipitoparietal Cortex. *J Neurosci, 40*(4), 917-931. doi:10.1523/JNEUROSCI.2700-19.2019

Gosseries, O., Yu, Q., LaRocque, J. J., Starrett, M. J., Rose, N. S., Cowan, N., & Postle, B. R. (2018). Parietal-Occipital Interactions Underlying Control- and Representation-Related Processes in Working Memory for Nonspatial Visual Features. *J Neurosci, 38*(18), 4357-4366. doi:10.1523/JNEUROSCI.2747-17.2018

Kok, P., Brouwer, G. J., van Gerven, M. A., & de Lange, F. P. (2013). Prior expectations bias sensory representations in visual cortex. *J Neurosci, 33*(41), 16275-16284. doi:10.1523/JNEUROSCI.0742-13.2013

Panichello, M. F., DePasquale, B., Pillow, J. W., & Buschman, T. J. (2019). Error-correcting dynamics in visual working memory. *Nat Commun, 10*(1), 3366. doi:10.1038/s41467-019-11298-3

Rademaker, R. L., Park, Y. E., Sack, A. T., & Tong, F. (2018). Evidence of gradual loss of precision for simple features and complex objects in visual working memory. *J Exp Psychol Hum Percept Perform, 44*(6), 925-940. doi:10.1037/xhp0000491

Taylor, R., & Bays, P. M. (2018). Efficient Coding in Visual Working Memory Accounts for Stimulus-Specific Variations in Recall. *J Neurosci, 38*(32), 7132-7142. doi:10.1523/JNEUROSCI.1018-18.2018
