# Supplementary material for: Delay-period activity in frontal, parietal, and occipital cortex tracks noise and biases in visual working memory
Source: PLoS Biol. 2020 Sep 8;18(9):e3000854. doi: 10.1371/journal.pbio.3000854 (PMC7500688; doi:10.1371/journal.pbio.3000854)
Supplement: Attachment — Submitted filename: DAMCorr_ResponseLetter_R2_Final.docx [file pbio.3000854.s010.docx]

**Point-by-point response to PBIOLOGY-D-20-00344R2**

We would like to thank the reviewers for their continued consideration of our manuscript. In particular, we thank Reviewer #3 for their thoughtful comments on how to contextualize and interpret our results. We have modified some of our claims in the manuscript, following the reviewer’s suggestions. We have also included one new paragraph in Discussion to address the reviewer’s concerns. Below we have reprinted the reviewers’ comments *in italicized Times New Roman*, and inserted our point-by-point responses in sans-serif Arial.

*Reviewer #1, Edward Ester: The authors have addressed my earlier comments re: the modeling approach to my satisfaction, and have done an admirable job responding to other reviewers' comments (including several new analyses and an analysis of third-party data to support their arguments). I have no further concerns and congratulate the authors on a nice study!*

Thanks very much!
 *Reviewer #2: I am happy with this version of the manuscript. The authors did a nice job of responding to the prior round of reviews. I have no remaining concerns. I feel that this work will have a substantial impact on the field.

Ed Vogel*

Thanks very much! *Reviewer #3, Paul Bays: See attachment.*

*I would like to thank the authors for their response to my previous comments. I have decided to unmask myself, as even if the authors haven’t figured out my identity already, I expect I will take this up with them next time I see them.*

We sincerely hope that this will be possible soon!

*In my original review, I raised the concern that - because delay interval was not manipulated in this study - the drift and diffusion parameters of the authors' dynamical model were simply measuring bias and variability in orientation estimation - factors that previous studies have shown are in fact largely static and unrelated to memory retention. The authors responded that they indeed could not distinguish between "influences of encoding and memory" in this study, but presented some fits of their model to data from a different study in which delay duration was manipulated. Their conclusion from this fitting is that "there’s indeed an effect of drift and diffusion during delay, albeit to a lesser degree [than during encoding]".*

*I was initially very unsure what to make of this response, so I looked up the previous study (Rademaker et al, 2018). Here are the results in question:*

*On the left, the biases that the authors’ model explain as temporal drift are indicated by deviation from the red line; the different shades of gray indicating delays between 1 and 12s. Clearly the vast majority of the bias is already present at the shortest time period (1 sec) and changes only minimally over the following 11 secs. This is confirmed by the plot on the right (AUC is a measure of bias strength). There are other studies showing the same thing and data collected in my lab shows it too, including in zero-delay conditions and set sizes > 1.*

*So if I have understood the authors’ response, they are claiming that ~90% of the temporal drift and diffusion their model identifies in that study did not occur during the 12s of memory retention but rather during the encoding period (the stimulus was presented for 200ms in the Rademaker study, although estimates of encoding are typically much briefer for 1 item). This seemed surprising, so I went back to the paper in which they had introduced the model on the basis of fitting it to color memory data (Panichello et al., 2019). I had actually read this paper before but have to admit this aspect had completely passed me by: in their fitted model the rate of drift during the exposure period has to be something like twenty times the rate during the delay to capture the data (in which presumably, like Rademaker’s data, biases were nearly constant over different delays).*

*So here’s the problem: the biases their model blames on “drift of memory representations towards stable attractors” really change very little over the memory delay. The authors say this is because the drift occurs during encoding not maintenance, but given the encoding period hasn’t been manipulated in any of these studies it’s really just supposition that anything dynamical is responsible, rather than e.g. some fixed bias and variability either in encoding or at retrieval. I would think the encoding period would be a time when memory representations were being driven towards the true stimulus orientation visible on the display – I certainly don’t see why they would be drifting away from it an order of magnitude faster than during the memory delay when there is no visible stimulus to anchor them. Maybe the authors have an explanation, but as far as I know they haven’t presented it anywhere yet, including this manuscript.*

*The authors also responded to my review with some analysis of the correlations between their parameters and BOLD measured at different timepoints – I’m not ignoring this, but I just don’t see it has much impact on this central point. The only way I can see for the authors to address this in the current manuscript is head on: make it abundantly clear from the abstract on down that the vast majority of the drift and diffusion their model claims to identify in this and their previous study doesn’t happen during retention in memory, and explain why they believe it must be taking place during encoding rather than there simply being some stable biases and variability in orientation perception that don’t evolve over time.*

*Paul Bays*

**Response**:

We thank the reviewer for clarifying his points raised in the previous review, which we believe we can now address in a more satisfactory and definitive manner. As we understand it, there are two major concerns:

1) If drift towards stable attractors changes very little over the memory delay, why is modeling of memory delay still necessary?
2) Given that most of the effects are at encoding and given the encoding period hasn't been manipulated in this experiment, why model behavior dynamically, rather than using some fixed bias and variability either in encoding or at retrieval?

First, we’d like to note that we feel the novel contribution of the present report is to relate these dynamical systems parameters to distinctive patterns of neural activity. We agree with the reviewer that the above concerns are important, and we believe we address them comprehensively below. However, the goal of the current manuscript is not to contrast the relative strength of dynamics in encoding/memory but rather to try to dissociate the neural basis for the two terms of the dynamical system model (drift and diffusion), regardless of whether they are stronger during encoding or memory.

Now, onto the substance of the review. Again, we thank the reviewer for providing such a clear articulation of his concerns. Below, we respond with a “head on” response, arguing for how our results are consistent with significant dynamics in both the encoding and memory periods and are consistent with previous reports from several different groups. That being said, we do also take the reviewer’s point that the attributability of estimates of drift to encoding versus delay should be addressed “from the abstract on down,” and we now do so in several places:

Abstract: we now make explicit that the estimates are from the “*whole trial*” (i.e., not attributable to a subcomponent of the task).

Introduction: on lines 64-66: “*Importantly, although the magnitude of drift is highest during memory encoding, its continued influence during the ensuing delay period is necessary to counteract the otherwise accumulating effect of random error (Panichello et al., 2019).”*

Results: on lines 103-105: *“Note that because memory delay was fixed in the current study, the DDM estimated drift and diffusion across both the encoding and memory delay periods.”*

Results: on lines 188-194: *“Because the length of memory delays was not manipulated in this fMRI study, we were limited to obtaining whole-trial behavioral measures of drift and diffusion that did not isolate encoding and delay contributions to these measures. Nonetheless, we were able to investigate whether the brain-behavior correlation changed dynamically over time by examining the temporal evolution of the within-subject correlation between BOLD activity and behavior (S5 Fig and S6 Fig), and also of the model performance of Model 1 and Model 2 (S7 Fig and S8 Fig).”*

Results: on lines 199-201: *“These dynamic changes suggested that our observations with BOLD-behavior correlations for drift and diffusion during delay cannot be interpreted in their entirety as encoding-related effects.”*

Discussion: There are several instances that are also applicable to more specific points of the review, and that are pasted in below.

With regard to point #1, i.e., why is modeling of memory delay still necessary? First, it is important to note that the drift and diffusion parameters accumulate over time. In the previous study that motivated the current one (Panichello et al., 2019), encoding was modeled as occurring over 1 s, while memory delay could be short (1 s) or long (7 s). We showed the increase in drift over the longer delay had an integrative effect on memory performance. Therefore, even though the magnitude of delay-period drift is less for the delay than for encoding, its impact is important because it accrues continuously over the entirety of the delay. Thus, despite the fact that the values of drift for the delay period were indeed ~20x smaller than those for encoding, they were nonetheless sufficient to explain the ~27% increase in mean absolute bias for the long versus the short delay-period trials (Panichello et al., 2019, Figure S1). We now make this point explicitly clear in the newly added paragraph of the Discussion section on lines 274-283:

*“Because previous behavioral work has suggested a substantially weaker influence of the delay period on memory performance, compared with encoding (Shin et al., 2017), one might ask whether the whole-trial measures reported here might also be dominated by encoding-related processes. Although overall magnitude of drift has, indeed, been shown to be higher during encoding [16], we nonetheless believe that delay-related processes had an important contribution to our estimates of drift and diffusion, for at least two reasons. First, because drift and diffusion during memory exert their effects continuously over time, their influence on behavior can substantially accumulate over the course of delay periods. For example, in the previous study (Panichello et al., 2019), delay-period drift has been shown to account for the increase in mean absolute bias on long versus short delay-period trials.”*

Furthermore, we think that there is a principled reason to expect drift to be weaker during the memory delay than during encoding. Drift scales with diffusion in an adaptive manner, to prevent random error from accumulating over the course of the memory delay. Therefore, the rate of diffusion is smaller during the memory delay than during encoding, because a smaller amount of drift is needed to work against this source of random error (which, like drift, accrues substantially over time; Panichello et al., 2019, Figure 7a). This is consistent with the remarkably low increase in error in working memory over time that has been reported by an independent group (Shin et al., 2017). We also make this point in the newly added paragraph on lines 283-287:

*“Second, from a functional perspective, drift counteracts the tendency of random diffusion to accumulate over time (Burak & Fiete, 2012; Panichello et al., 2019), and this countervailing influence of drift should scale with the rate of diffusion. Indeed, the previous study shows the lower drift rate during memory is sufficient to substantially prevent the accumulation of random errors over time (Panichello et al., 2019).”*

The reviewer noted that biases increase relatively little over time in the Rademaker et al. (2018) data. This is to be expected because that study used a low load (load = 1) which was previously observed to generate weaker dynamics than trials with higher loads. In that sense, their study provides a lower bound on the degree to which bias increases over time.

And so, although we agree with the reviewer that drift is weaker during the memory delay relative to encoding, we believe this does not obviate the need to model behavior dynamically as it allows us to ask a new question – where in the brain is neural activity correlated with drift and diffusion?

Now for point #2. The important question raised by the reviewer is: how can we model memory dynamically, if the encoding period isn’t manipulated (e.g., by varying duration of stimulus presentation)?

First, we would like to clarify that our encoding term is not meant to capture the full dynamics of encoding that unfold during visual processing. Instead, we aim to capture the biases and noise that the encoding process introduces into memories. In other words, we hope to capture the same biases the reviewer has explained using principles of efficient coding (Taylor & Bays, 2018). We view the value of our approach as: (1) it appropriately treats encoding as a dynamic (i.e., not instantaneous) process and (2) it can parsimoniously describe both the outcome of encoding dynamics (as evidenced by the ability of the model to accurately capture reports following short memory delays) and memory dynamics using the same framework. In particular, this second point is non-trivial and suggests a common mechanism.

Furthermore, given that the encoding period was fixed across conditions, we only need to vary the duration of the memory delay to isolate the dynamics during encoding (which will be fixed ‘costs’) and the memory delay (which will vary as a function of the delay length). This was our approach in the previous study. Importantly, as noted above, the previous work showed that the same drift and diffusion framework was capable of capturing the distribution of reports at both encoding and delay. Introducing a separate drift function during the encoding period did not increase the explanatory power of the model (unpublished result). It is for this reason that we think it is reasonable to use a ‘whole-trial’ fit to estimate drift/diffusion and, then, correlate these terms with activity in different brain regions.

We agree with the reviewer that multiple processes may be at play during the encoding period, and it would be interesting to vary encoding periods to tease these apart in future studies. For example, in one study from the reviewer’s group (Bays, Gorgoraptis, Wee, Marshall, & Husain, 2011), they varied encoding duration from 25 ms to 1000 ms, and demonstrated an initial rapid rise in precision with increasing encoding duration. This initial rate decreased as memory load increased, and memory precision saturated at around 200-300 ms for all memory loads. It would be interesting to investigate, for example, how drift and diffusion would change with insufficient encoding time (e.g., < 100 ms). However, addressing this question is outside the scope of the current study, because the purpose of the current study is to relate diffusion and drift to *delay-period activity* in frontoparietal and occipital cortex. Therefore, the future result of asking how encoding time affects drift/diffusion should not change the interpretation of our current results.

Lastly, we think the neural data can also, at least in part, address the reviewer’s core concern. In the previous revision we added an analysis by plotting the full time course of correlations between BOLD activity and behavioral parameters of drift and diffusion. This analysis, still included in this second revision, shows that the whole-trial measures of drift and diffusion relate most strongly to the neural data during the memory delay. Thus, although we are not able to disentangle encoding and memory effects with the behavioral data alone, the correlations with neural data clearly suggest an important contribution of delay-period effects, which is consistent with the behavioral results in the previous study (Panichello et al., 2019) that provided the impetus for the present work.

**References**

Bays, P. M., Gorgoraptis, N., Wee, N., Marshall, L., & Husain, M. (2011). Temporal dynamics of encoding, storage, and reallocation of visual working memory. *J Vis, 11*(10). doi:10.1167/11.10.6

Burak, Y., & Fiete, I. R. (2012). Fundamental limits on persistent activity in networks of noisy neurons. *Proc Natl Acad Sci U S A, 109*(43), 17645-17650. doi:10.1073/pnas.1117386109

Panichello, M. F., DePasquale, B., Pillow, J. W., & Buschman, T. J. (2019). Error-correcting dynamics in visual working memory. *Nat Commun, 10*(1), 3366. doi:10.1038/s41467-019-11298-3

Rademaker, R. L., Park, Y. E., Sack, A. T., & Tong, F. (2018). Evidence of gradual loss of precision for simple features and complex objects in visual working memory. *J Exp Psychol Hum Percept Perform, 44*(6), 925-940. doi:10.1037/xhp0000491

Shin, H., Zou, Q., & Ma, W. J. (2017). The effects of delay duration on visual working memory for orientation. *J Vis, 17*(14), 10. doi:10.1167/17.14.10

Taylor, R., & Bays, P. M. (2018). Efficient Coding in Visual Working Memory Accounts for Stimulus-Specific Variations in Recall. *J Neurosci, 38*(32), 7132-7142. doi:10.1523/JNEUROSCI.1018-18.2018
